# NKX6.3 modulation of mitotic dynamics and genomic stability in gastric carcinogenesis

**DOI:** 10.1186/s12964-025-02030-4

**Published:** 2025-01-20

**Authors:** Jung Hwan Yoon, Jeong-Kyu Kim, Jung Woo Eun, Hassan Ashktorab, Duane T. Smoot, Suk Woo Nam, Won Sang Park

**Affiliations:** 1 Department of Pathology, Functional RNomics Research Center, College of Medicine, The Catholic University of Korea, 222 Banpo-daero, Seocho-gu, Seoul, 06591 South Korea; 2 Department of Life Science, Chung-Ang University, Seoul, South Korea; 3 Department of Gastroenterology, Ajou University School of Medicine, Suwon, South Korea; 4 Department of Medicine, Howard University, District of Columbia, Washington, 20060 USA; 5 Department of Medicine, Meharry Medical Center, Nashville, TN 37208 USA

**Keywords:** NKX6.3, Mitotic integrity, Genomic stability, Gastric carcinogenesis

## Abstract

**Background:**

Gastric cancer remains a significant global health challenge, characterized by poor prognosis and high mortality rates. Mitotic integrity and genomic stability are crucial in maintaining cellular homeostasis and preventing tumorigenesis. The transcription factor NKX6.3 has emerged as a potential regulator of these processes in gastric epithelial cells, prompting an investigation into its role in gastric cancer development.

**Methods:**

We employed a combination of in vitro and in vivo techniques to elucidate the impact of NKX6.3 depletion on mitotic dynamics and genomic stability in gastric epithelial cells. Quantitative real-time PCR and Western blot analyses were conducted to assess the expression of mitosis-related genes and proteins. Flow cytometry was utilized to evaluate cell cycle distribution, while immunofluorescence microscopy enabled the visualization of mitotic abnormalities. Statistical analyses, including Student’s t-test and ANOVA, were performed to determine the significance of our findings.

**Results:**

Our results demonstrate that NKX6.3 depletion leads to significant mitotic defects, characterized by increased chromosome misalignment and lagging chromosomes during anaphase. These abnormalities corresponded with elevated levels of genomic instability markers, indicating compromised genomic integrity. Furthermore, the loss of NKX6.3 resulted in altered expression of key regulatory proteins involved in mitosis and DNA repair pathways, suggesting a mechanistic link between NKX6.3 and the maintenance of genomic stability in gastric epithelial cells. Depletion of NKX6.3 resulted in accelerated cell cycle progression and the formation of abnormal mitotic figures, leading to genomic instability characterized by increased DNA content and structural abnormalities. In both in vitro and xenograft models, the depletion of NKX6.3 significantly upregulated AurkA and TPX2, which correlated with gains in DNA copy number. An inverse relationship was observed between NKX6.3 expression and the levels of AurkA and TPX2 in human gastric cancer tissues.

**Conclusions:**

This study highlights the essential role of NKX6.3 in regulating mitotic integrity and genomic stability in gastric carcinogenesis. The findings suggest that targeting NKX6.3 may offer a novel therapeutic strategy for improving treatment outcomes in gastric cancer by restoring mitotic fidelity and genomic stability.

**Trial registration:**

This study was not registered.

**Supplementary Information:**

The online version contains supplementary material available at 10.1186/s12964-025-02030-4.

## Background

Gastric cancer (GC) is a significant global health issue, ranking as the fifth most prevalent cancer and the third highest cause of cancer-related deaths worldwide [1]. Although there have been advancements in diagnosis and treatment, the prognosis for patients with advanced GC remains discouraging. Consequently, it is crucial to urgently enhance our understanding of the molecular mechanisms underlying tumor development and progression.

Cell cycle dysregulation, particularly aberrant mitotic processes, plays a pivotal role in the development and progression of GC [2–4]. Mitosis, the process of cell division, is tightly regulated to ensure accurate chromosome segregation and genomic stability [5, 6]. Disruption of mitotic fidelity can lead to chromosomal instability (CIN), a hallmark of cancer characterized by aneuploidy and structural chromosomal aberrations [7, 8]. CIN is recognized as a driver of tumorigenesis because it can cause genetic alterations and functional imbalances that lead to uncontrolled cell growth and survival [9–11]. CIN not only impacts tumor heterogeneity but also promotes the emergence of oncogenic mutations and resistance to therapy. As a result, this ultimately leads to tumor progression and metastasis.

NKX6.3, a member of the NKX6 subfamily located on chromosome 8p11.21, encodes a 266-amino acid protein that is specifically expressed in the distal stomach epithelium, where it localizes to the lower/base region of the gastric unit [12, 13]. Its primary significance lies in the regulation of cellular processes crucial for development, differentiation, and tissue homeostasis. Recent research has indicated that NKX6.3 modulates DNA replication stress and subsequent DNA damage, which in turn regulates the efficacy of DNA repair mechanisms in the context of GC [14]. Despite emerging evidence of NKX6.3’s involvement in GC progression [14–16], the precise molecular mechanisms by which it exerts its tumor-suppressive effects - especially regarding cell cycle dynamics - have yet to be thoroughly investigated.

This thesis aims to further investigate how NKX6.3 inactivation contributes to gastric carcinogenesis, emphasizing its unique role in the maintenance of chromosomal stability. By elucidating the pathways through which NKX6.3 prevents CIN in gastric epithelial cells by regulating cell cycle progression and ensuring mitotic fidelity, this research not only addresses a significant knowledge gap but also highlights its potential as a diagnostic biomarker and therapeutic target of GC. ​.

## Materials and methods

### Samples and ethics approval

The Chonnam National University Hwasun Hospital Biobank, a member of the Korea Biobank Network, provided a total of 65 frozen human GC tissues. This study received approval from the Institutional Review Board of The Catholic University of Korea, College of Medicine (MC15SISI0015). Written informed consent was obtained from all subjects in accordance with the Declaration of Helsinki. There was no evidence of familial cancer in any of the patients. Animal experiments were performed in mice maintained under pathogen-free conditions after approval by the Animal Experiment Ethics Committee of the Catholic University of Korea College of Medicine (CUMC-2017-0018-01).

### Cell culture and transfection

A non-neoplastic gastric epithelial cell line called HFE-145 was cultured at 37^o^C in 5% CO_2_ using DMEM medium supplemented with 10% heat-inactivated fetal bovine serum. In addition, human primary stomach epithelial cells (PSEC; Cat No. H-6039) from Cell Biologics were cultured using Complete Epithelial Cell Medium w/ Kit (Cat No. H-6621). The shNKX6.3 was cloned into the pGFP-C-shLenti vector produced by Origene (Rockville, MD, USA; Cat. No. TL319045). The HFE-145 and PSEC cells were then transfected with shNKX6.3 using Lipofectamine Plus transfection reagent (Invitrogen, Carlsbad, CA, USA; Cat. No. L3000015), following the manufacturer’s instructions.

We generated stable NKX6.3 knockdown cells in two different target sites, HFE-145^shNKX6.3#1^ and HFE-145^shNKX6.3#2^, as well as non-targeting shRNA transfectant HFE-145^shCtrl^ cells, as described previously [14]. PSEC cells were transiently transfected with either shRNA targeting NKX6.3 (designated as PSEC^shNKX6.3^) or non-targeting shRNA (designated as PSEC^shCtrl^). The effective knockdown of NKX6.3 was verified through Western blot and ELISA analyses in both stably transfected HFE-145 cells and transiently transfected PSEC cells.

### In vivo Xenograft mouse experiment

For the in vivo xenograft assay, 5 × 10^6^ HFE-145^shCtrl^, HFE-145^shNKX6.3#1^, and HFE-145^shNKX6.3#2^ cells were mixed with 0.2 ml of phosphate-buffered saline (PBS, pH7.4) and 30% (v/v) Matrigel (BD Biosciences, San Jose, CA, USA; Cat. No. 356230). BALB/c-nude mice between four and five weeks old were purchased from ORIENT (Seongnam, Korea). The cells were injected subcutaneously into the side of the flank of the nude mice. Tumor formation at the injection site was monitored twice a week. Tumor volumes were measured every three days, starting from the third week, using the formula length × width^2^ × 0.5. After seven weeks, the tumors were removed, photographed, and weighed. Each group consisted of five mice, and tumor growth was quantified by measuring the tumor sizes with calipers.

### Synchronization and cell cycle analysis

HFE-145^shCtrl^, HFE-145^shNKX6.3#1^, and HFE-145^shNKX6.3#2^ cells were treated with nocodazole (200 ng/mL; Sigma-aldrich, St Louis, MO, USA; Cat. No. SML1665) for 0, 3, 6, 9, 12, 15, and 18 h to create synchronized populations. The cells were harvested at different times after synchronization and processed for immunoblotting analysis. For flow cytometric analyses, the HFE-145^shCtrl^, HFE-145^shNKX6.3#1^, and HFE-145^shNKX6.3#2^ cells were treated with 300 ng/mL of nocodazole. They were then harvested at various times after treatment, fixed, and stained with propidium iodide (Sigma-aldrich; Cat. No. 81845). The DNA contents of 10,000 cells per sample were analyzed using a BD FACSCanto II (BD Biosciences) and further analyzed with FlowJo software (v.10.10.0; BD, Ashland, OR, USA).

### Mitotic index

Mitotic HFE-145^shCtrl^, HFE-145^shNKX6.3#1^, HFE-145^shNKX6.3#2^, PSEC^shCtrl^, and PSEC^shNKX6.3^ cells were fixed in 3.7% formaldehyde for 30 min at 37 °C. Then, they were permeabilized with ice-cold methanol for 20 min. After washing with PBS, the cells were incubated at room temperature for 20 s with 1 µg/mL DAPI (Sigma-Aldrich; Cat. No. D9542). The coverslips were mounted in 50% glycerol in phosphate-buffered saline (PBS) containing 1 mg/mL ascorbic acid. Finally, the cells were examined under a Carl Zeiss LSM800 w/Airyscan confocal microscope using a 100× objective (Carl Zeiss Microscopy GmbH, Jena, Germany). The images were analyzed with ImageJ software. The mitotic index was calculated as the percentage of mitotic cells out of 100 cells counted per experiment.

### Metaphase spread

To prepare the chromosome, the HFE-145^shCtrl^, HFE-145^shNKX6.3#1^, HFE-145^shNKX6.3#2^, PSEC^shCtrl^, and PSEC^shNKX6.3^ cells were first treated with 50 ng/mL colcemid (Sigma-Aldrich; Cat. No. 234109) for 120 min. After that, they were incubated at 37 °C for 18 min in a solution containing 0.075 mol/L potassium chloride. Following this, the Carnoy’s fixative (3:1, methanol: acetic acid) was added drop by drop. The cells were then incubated in fixative for 1 h, pelleted at 1,000 ×g, and replenished with more fixative. Next, the cells were incubated at 4 °C overnight, with the fixative being replenished. The fixed cells were placed on uncoated microscope slides and allowed to dry at room temperature for at least 24 h. The slides were then stained with Giemsa staining solution (Sigma Aldrich; Cat. No. 32884) for 4 min. Finally, the slides were analyzed for total gaps and breaks under 100× magnification using a Carl Zeiss Axioimager M1 microscope (Carl Zeiss Microscopy). A total of 100 metaphases were scored for each sample, with 50 metaphases being scored in two independent experiments.

### Whole genome sequencing and copy number data analysis

Copy Number Alterations (CNAs) were analyzed using whole-genome sequencing (WGS), as previously described [15]. In brief, 0.5–3 µg of DNA from the HFE-145^shCtrl^, HFE-145^shNKX6.3#1^, and HFE-145^shNKX6.3#2^ cells were utilized to prepare the sequencing library. This was achieved by shearing the DNA and then ligating the sequencing adaptors. Subsequently, the captured DNA was sequenced on the Illumina HiSeq X Ten platform (Illumina, San Diego, CA, USA), generating paired-end sequencing reads for the HFE-145^shCtrl^, HFE-145^shNKX6.3#1^, and HFE-145^shNKX6.3#2^ cells. The quality of the sequence reads was assessed using FastQC 0.1 software. The paired-end library, consisting of approximately 500-bp inserts, was sequenced with 150-bp paired-end reads. The raw sequencing data were processed using an ultrafast Isaac DNA sequence aligner, which was designed to align next-generation sequencing data with a low-error rate. The human genome hg19, GRCh37 was used as a reference for alignment.

The WGS data were analyzed using Nexus Copy Number version 9.0 software (Biodiscovery Inc., El Segundo, CA, USA). The Nexus calling algorithm, known as SNPrank segmentation, is based on the Circular Binary Segmentation model. Abnormal copy number variants and allele ratios were determined using Log2 ratio values. Values greater than 0.2 were considered gains, while values less than − 0.2 were considered losses. Thresholds for identifying high copy number gains and homozygous deletions were set at 0.65 and − 0.65, respectively.

After extracting genomic DNA extracted from the HFE-145^shCtrl^, HFE-145^shNKX6.3#1^, and HFE-145^shNKX6.3#2^ cells, we conducted a real-time SYBR Green quantitative polymerase chain reaction (qPCR) using a Bio-Rad IQ5 real-time PCR platform (Bio-Rad Laboratories, Hercules, CA, USA). We designed specific primers based on GenBank’s genomic sequence for analyzing the DNA copy numbers of *AurkA* and *TPX2*. To normalize the samples, we performed PCR amplification and used oligonucleotide primers specific to constitutively expressed genes and glyceraldehyde-3-phosphate dehydrogenase (GAPDH). The primers for SYBR Green analysis were designed using gene-specific non-homologous DNA sequences. The primer sequences can be found in Supplementary Table S1.

### Transcriptome analysis

The total RNA of HFE-145^shCtrl^, HFE-145^shNKX6.3#1^, and HFE-145^shNKX6.3#2^ cells was extracted using TRIzol reagent (Invitrogen; Cat. No. 15596026) and assessed for quality. Only RNA samples with RNA integrity number values above 8 were used for transcriptome sequencing on the Illumina HiSeq 2500 platform (Illumina). Library preparation involved mRNA selection, fragmentation, cDNA synthesis, adapter ligation, and PCR amplification. After evaluation and pooling of the libraries, sequencing was performed. The raw reads were trimmed, filtered, aligned to the mouse reference genome (GRCm38), and quantified to determine gene expression levels using Fragment Per Kilobase of transcript per Million mapped reads. Differentially expressed genes (DEGs) were identified using DESeq2 with specified cutoffs. These DEGs were then subjected to gene set enrichment analysis (GSEA) to identify enriched gene sets associated with inflammation, immune response, and mitochondria. GSEA was performed using the pre-ranked mode, and the results were visualized using EnrichmentMap in Cytoscape. A comprehensive gene network analysis was conducted using Genemania to identify the hub gene among the 22 candidate genes associated with the mitotic spindle. Each gene was represented as a node, with edges depicting interactions and the thickness of the edges indicating the strength of the association. The hub gene, which played a central role in the network and exhibited significant associations, was identified by analyzing connectivity and centrality measures, particularly the weighted degree of interactions.

### Fluorescence in situ hybridization

Interphase fluorescence in situ hybridization (FISH) was performed according to the description [15]. Bacterial artificial chromosomes were obtained from the BACPAC (Oakland, CA, USA) and probes were prepared as described [15]. Specifically, gene locus-specific probes and chromosome 20-specific probes were used to detect *AurkA* and *TPX2*. The integrity and accurate localization of all probes were confirmed by hybridizing them to the HFE-145^shCtrl^, HFE-145^shNKX6.3#1^, and HFE-145^shNKX6.3#2^ cells. The slides were examined using an ImagingZ1 microscope (Carl Zeiss).

### Co-immunoprecipitation

Co-immunoprecipitations were performed as follows. Cells from a nearly confluent 10 cm dish were collected using 1 ml of protein lysis buffer, following the method described earlier. Next, bovine serum albumin (BSA)-blocked protein G beads (Fastflow; Millipore-Sigma; Cat. No. 16–201) were added and incubated at 4 °C for 30 min to remove any unwanted substances from the lysates. A 1% portion of the total mixture was then separated and rotated for 1 h at 4 °C in the presence of 2 µg of anti-AurkA and BSA-blocking protein G beads to enable immunoprecipitation. After washing the beads with lysis buffer, the proteins were extracted using Laemmli loading buffer, and immunoblots were conducted as previously described [15].

### Real-time RT-qPCR

RNA was extracted from the cells using the RNeasy kit (Qiagen, Valencia, CA, USA; Cat. No. 74104). The extracted RNA was then converted to cDNA reverse transcription kit (Applied Biosystems, Carlsbad, CA, USA; Cat. No. 4368814). For quantification, qPCR was conducted following standard protocols on a Bio-Rad IQ5 real-time PCR platform (Bio-Rad). As a control, the average mRNA expression value in non-neoplastic gastric tissues was used. The mRNA expression change in each case was subsequently normalized to the mean value in non-neoplastic gastric tissues. A decrease or increase in mRNA expression was indicated by changes less than 0.5-fold or greater than 1.5-fold, respectively. All primer details can be found in the Supplementary Table S1.

### Immunoblotting and direct ELISA

The effects of NKX6.3 on the expression of AurkA, TPX2, and cell cycle-regulators were determined in the HFE-145^shCtrl^, HFE-145^shNKX6.3#1^, HFE-145^shNKX6.3#2^, PSEC^shCtrl^, and PSEC^shNKX6.3^ cells, and xenograft tumor tissues by immunoblots as previously described [14]. Briefly, the cells, mice, and human gastric tissues were washed twice with cold PBS. They were then lysed in RIPA buffer, which contained 50 mM Tris pH 7.4, 150 mM NaCl, 1% NP-40, 0.5% sodium deoxycholate, 0.1% sodium dodecyl sulfate (SDS), 1 mM phenylmethyl sulfonyl fluoride, and a complete protease and phosphatase inhibitor cocktail (Roche, Indianapolis, IN, USA; Cat. No. 11836170001). The lysates were collected by scraping after 5 min and cleared by centrifugation at 12,000 rpm for 10 min. Protein quantification was performed using the BCA Assay, and the sample proteins were separated by SDS-polyacrylamide gel electrophoresis. The proteins were then transferred from the gels to polyvinylidene fluoride membranes (Bio-Rad). After blocking the membranes in 5% BSA-Tris-buffered saline Tween 20 for 30 min at room temperature, they were incubated overnight with the indicated primary antibodies at 4 °C. Following this, the membranes were washed three times, incubated with peroxidase-conjugated secondary antibodies (Sigma, St. Louis, MD, USA), and the immunoreactions were detected by enhanced chemiluminescence (Millipore, Billerica, MA, USA; Cat. No. WBULP-10ML) on a LAS-4000.

To confirm the effects of NKX6.3 on the expression of AurkA, TPX2, and cell cycle-regulators, the HFE-145^shCtrl^, HFE-145^shNKX6.3#1^, HFE-145^shNKX6.3#2^, PSEC^shCtrl^, and PSEC^shNKX6.3^ cells were analyzed using direct ELISA. Briefly, the cell lysates in PBS were coated on 96-well microtiter plates and then blocked with 1% BSA for 1 h at 37 °C. After washing, each primary detection antibody was added at a dilution of 10,000–20,000× in blocking buffer, followed by incubation at 37 °C for 1 h. Subsequently, an HRP-conjugated secondary antibody was added at a dilution of 15,000× in blocking buffer and incubated for 1 h at 37 °C. After another washing step, TMB substrate solution was added, and the plates were incubated at 37 °C for 20 min in the dark. The enzyme reaction was stopped by adding the stop solution, and absorbance was measured at 450 nm using a microplate reader for data analysis. All antibodies are described in the Supplementary Table S2.

### Immunohistochemistry

For the immunohistochemical analysis, xenograft tumor nodule and tissue microarray recipient blocks were constructed containing 157 GC tissues from formalin-fixed paraffin embedded specimens. Three tissue cores from each cancer (2 mm in diameter) were taken and placed in a new recipient paraffin block using a commercially available microarray instrument (Beecher Instruments, Micro-Array Technologies, Silver Spring, MD, USA), according to established methods. One cylinder containing normal gastric mucosa next to each tumor was also transferred to the recipient block. 2 μm sections were cut the day before use and stained according to standard protocols.

To enhance the immunohistochemistry signal, two methods were employed. Firstly, antigen retrieval was performed in citrate buffer, as well as signal amplification using biotinylated tyramide, as previously described [14]. The sections were then incubated overnight at 4 °C with NKX6.3, AurkA, TPX2, and Ki-67 antibodies (1/100). Detection was carried out using biotinylated goat anti-rabbit antibodies (Sigma-Aldrich; Cat. No. A3687), followed by incubation with a peroxidase-linked avidin-biotin complex. Diaminobenzidine was used as a chromogen, and the slides were counterstained with Mayer’s hematoxylin. Staining for NKX6.3, AurkA, TPX2, and Ki-67 antigen was considered positive when more than 30% of the nucleus was positively stained. The results were independently reviewed by two pathologists. For the negative controls, primary antibodies were replaced with non-immune serum.

### Statistical analyses

The MTT, BrdU, FACS, ELISA, real-time qPCR, and Western blot assays were each performed independently at least three times. The results are presented as means ± SD in the bar graphs. Differences between the means were assessed using one- or two-way ANOVA. Statistical analyses were conducted using GraphPad Prism version 7.00 for Windows (GraphPad Software, La Jolla, CA, USA). The student’s t-test was used to analyze all other data. Statistical significance is indicated by asterisks in the figures: * *P* ≤ 0.05, ** *P* ≤ 0.01, *** *P* ≤ 0.001 and **** *P* ≤ 0.0001.

## Results

### Depletion of NKX6.3 drives aberrant cell cycle acceleration

**Fig. 1:**
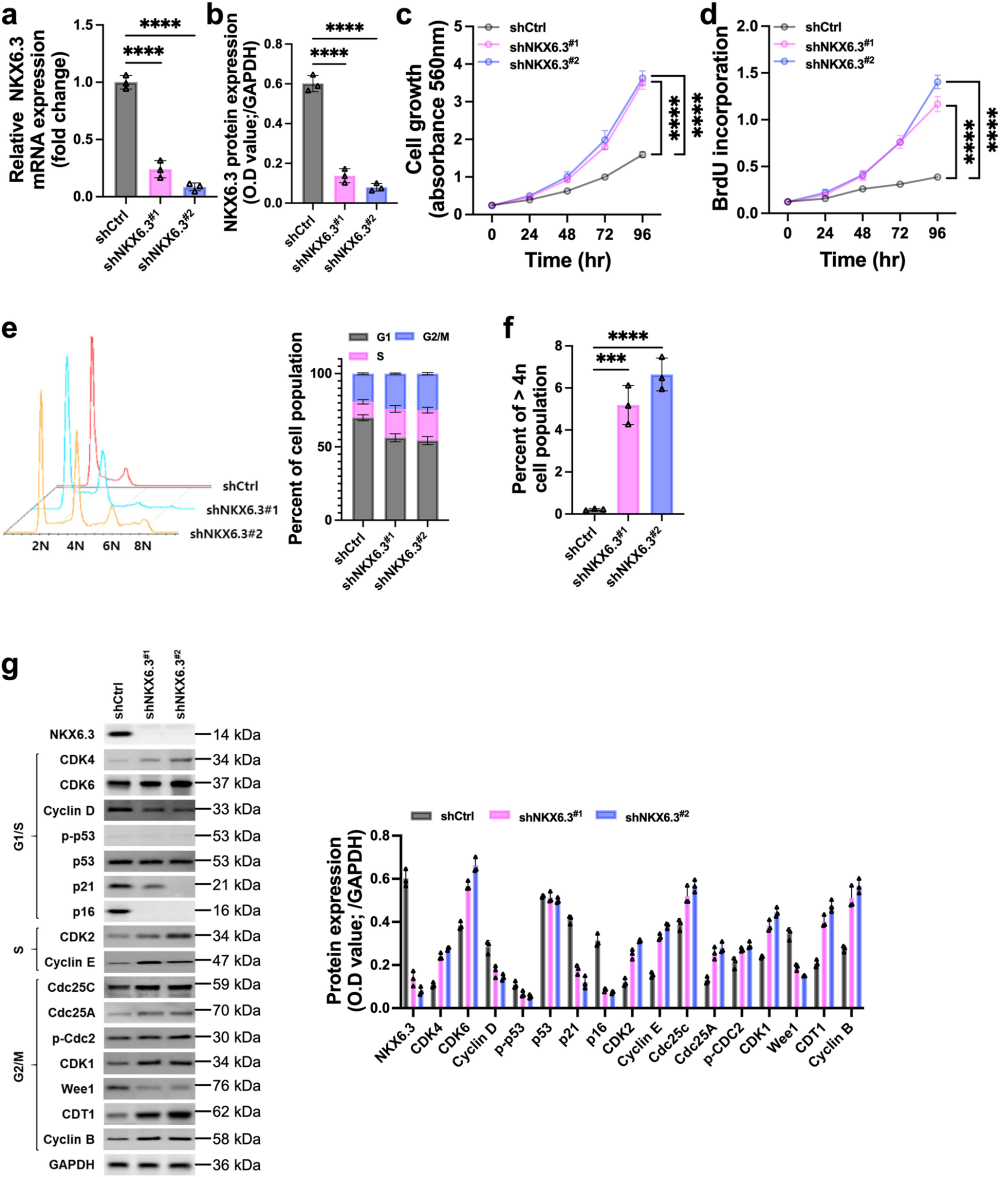
Depletion of NKX6.3 disrupts cell cycle progression and promotes aneuploidy in HFE-145 gastric epithelial cells. **a** and **b** mRNA (**a**) and protein (**b**) expression levels of NKX6.3 in HFE-145 cells following stably shRNA-mediated depletion. (**c** and **d**) Cell growth (**c**) and cell proliferation (**d**) assays of HFE-145^shCtrl^, HFE-145^shNKX6.3#1^ and HFE-145^shNKX6.3#2^. MTT and BrdU incorporation assays were measured at indicated time points after seeding. **e** Cell cycle distribution of HFE-145^shCtrl^, HFE-145^shNKX6.3#1^ and HFE-145^shNKX6.3#2^. Propidium iodide staining and flow cytometry were used to analyze cell cycle distribution. **f** Percentage of aneuploid cells in HFE-145^shCtrl^, HFE-145^shNKX6.3#1^ and HFE-145^shNKX6.3#2^. Flow cytometry analysis identified cells with DNA content exceeding the 4 N threshold. **g** Western blot and ELISA analysis of cell cycle regulatory proteins in HFE-145^shCtrl^, HFE-145^shNKX6.3#1^ and HFE-145^shNKX6.3#2^. Protein expression levels of Wee1, p21, p16, CDK1, p-Cdc2, cyclin E, and cyclin B were analyzed. GAPDH served as a loading control. All data are presented as mean ± SD of three independent experiments. Asterisks indicates *** *p* < 0.001; **** *p* < 0.0001

Our study highlights the crucial role of NKX6.3 in regulating cellular proliferation and genomic stability in HFE-145 normal gastric epithelial cells. The targeted reduction of NKX6.3 using shRNA resulted in a significant decrease in both mRNA and protein expression, prompting a detailed functional investigation (Fig. 1a and b). We observed a marked increase in cell growth and proliferation as early as 48 h post-depletion (Fig. 1c and d), underscoring NKX6.3 as a key regulator and checkpoint in the cell cycle. Depletion of NKX6.3 disrupted normal cell cycle progression, with a notable reduction in the G1 phase and increases in the S and G2/M phases, leading to aneuploid cells exceeding the 4 N DNA content threshold (Fig. 1e and f). Western blot analysis revealed decreased levels of G1 phase regulators (Wee1, p21, and p16) and increased levels of S and G2/M phase drivers (CDK1, p-Cdc2, cyclin E, and cyclin B), indicating accelerated cell cycle progression in NKX6.3-depleted cells (Fig. 1g). These findings underscore the critical role of NKX6.3 in maintaining cell cycle integrity and preventing genomic instability, both of which are essential processes in the proliferation of gastric epithelial cells and tumorigenesis.

### Temporal analysis of cell cycle and protein expression following nocodazole treatment in HFE-145 cells

**Fig. 2:**
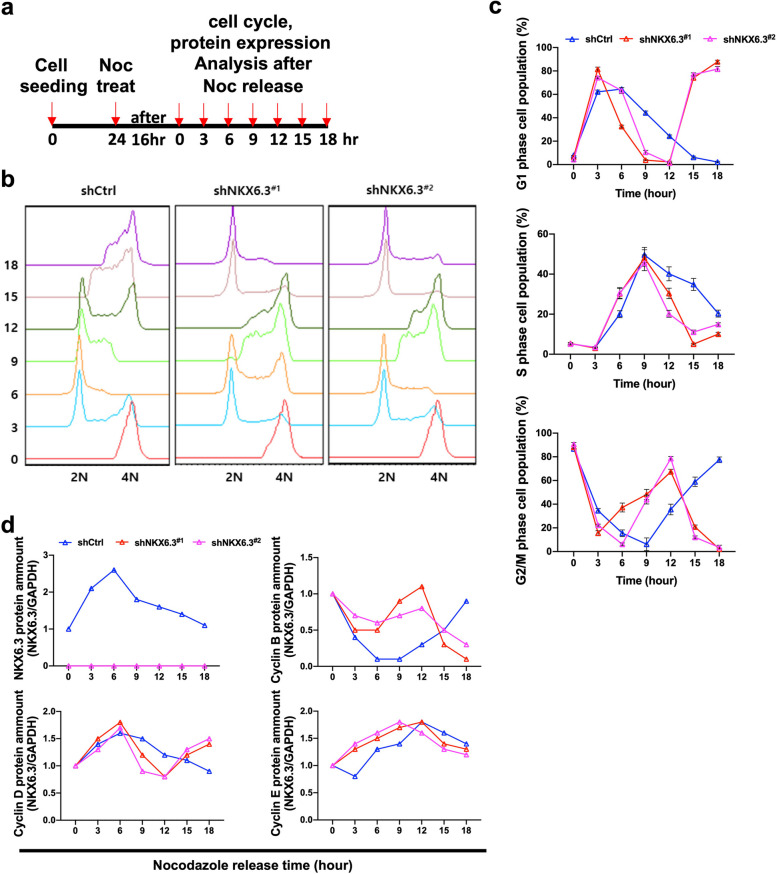
Temporal analysis of cell cycle and protein expression following nocodazole treatment in NKX6.3-depleted HFE-145 cells. **a** Schematic representation of the experimental design depicting the time-course experiment following nocodazole treatment in HFE-145^shCtrl^, HFE-145^shNKX6.3#1^ and HFE-145^shNKX6.3#2^. Cells were synchronized at mitosis using a nocodazole block and then released. Cell cycle distribution and protein expression were analyzed at various time points post-release. **b** and **c** Cell cycle distribution of HFE-145^shCtrl^, HFE-145^shNKX6.3#1^ and HFE-145^shNKX6.3#2^ cells following nocodazole release. Cell cycle distribution was analyzed by flow cytometry at the indicated time points. Representative flow cytometry plots (**b**) and quantification of the percentage of cells (**c**) in each cell cycle phase. **d** ELISA analysis of cyclin B, D, and E protein expression levels in HFE-145^shCtrl^, HFE-145^shNKX6.3#1^ and HFE-145^shNKX6.3#2^ cells following nocodazole release. Protein expression was analyzed at the indicated time points. GAPDH served as a loading control

To investigate how cells respond to NKX6.3 depletion, we conducted a time-course experiment using HFE-145 cells exposed to nocodazole. Cells were collected at 0, 3, 6, 9, 12, 15, and 18 h after nocodazole release to observe changes in the cell cycle and protein expression (Fig. 2a). We also synchronized HFE-145^shCtrl^, HFE-145^shNKX6.3#1^, and HFE-145^shNKX6.3#2^ cells at mitosis using nocodazole, followed by staggered release. NKX6.3-depleted cells exhibited accelerated cell cycle progression compared to HFE-145^shCtrl^ cells, with a rapid increase in the S phase population post-release (Fig. 2b and c). Furthermore, the levels of cyclins B, D, and E were found to correlate with the accelerated cell cycle progression (Fig. 2d), indicating a direct relationship between NKX6.3 depletion and enhanced cell cycle dynamics.

### NKX6.3 depletion amplifies mitotic anomalies and genomic instability

**Fig. 3:**
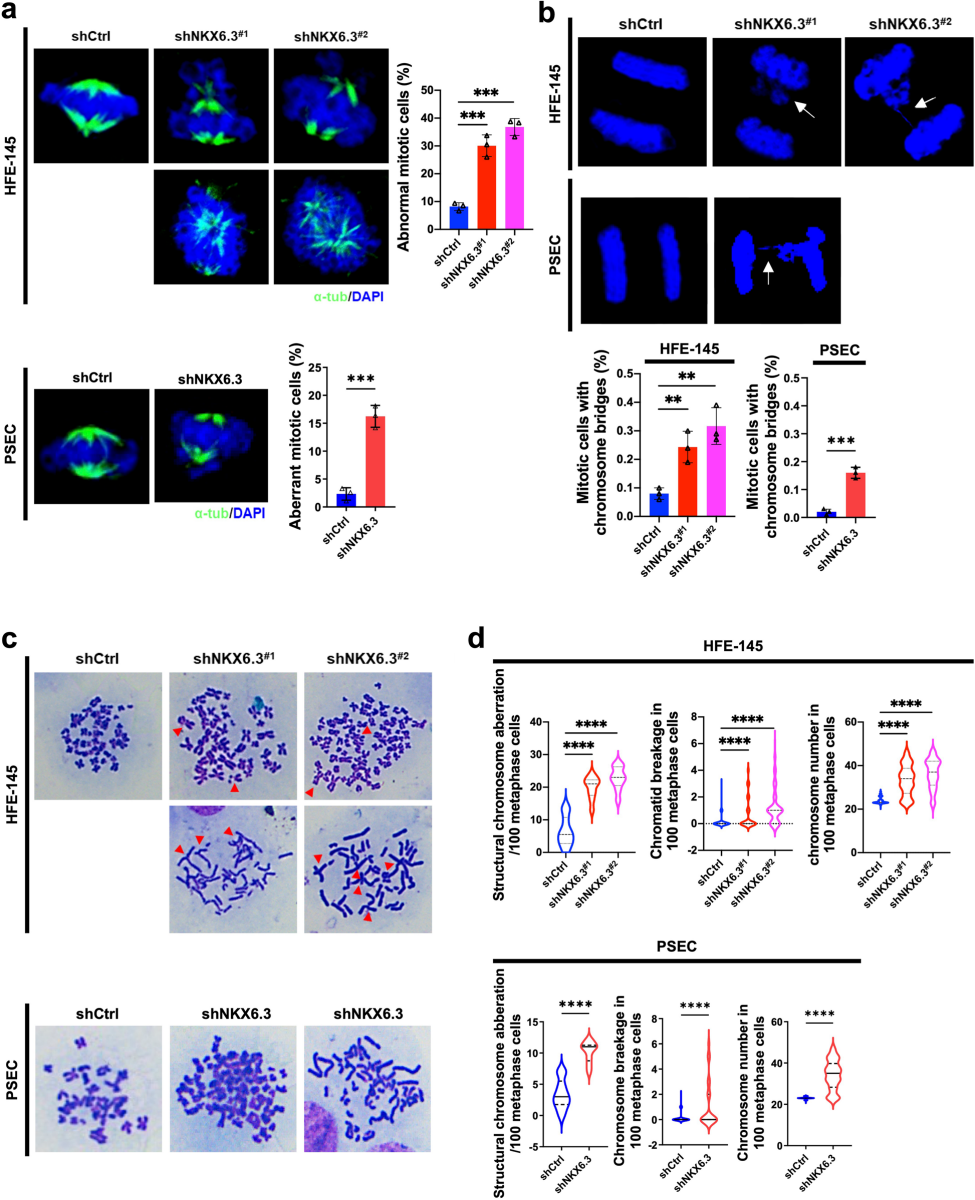
NKX6.3 depletion induces mitotic abnormalities and chromatin disruptions.** a** Representative microscopic images and quantification of abnormal mitotic cells showing irregular α-tubulin/DAPI staining patterns in HFE-145^shCtrl^, HFE-145^shNKX6.3#1^, HFE-145^shNKX6.3#2^, PSEC^shCtrl^, and PSEC^shNKX6.3^ cells. Cells were stained with α-tubulin (green) to visualize mitotic spindles and DAPI (blue) for DNA. Scale bar, 10 μm. **b** Representative microscopic images and quantification of chromosome bridge formation during mitosis in HFE-145^shCtrl^, HFE-145^shNKX6.3#1^, HFE-145^shNKX6.3#2^, PSEC^shCtrl^, and PSEC^shNKX6.3^ cells. **c** Analysis of structural deformities and chromatin condensation in HFE-145^shCtrl^, HFE-145^shNKX6.3#1^, HFE-145^shNKX6.3#2^, PSEC^shCtrl^, and PSEC^shNKX6.3^ cells, indicating significant differences. **d** Violin plots depicting the distribution of the structural deformation ratio and chromatin condensation index in HFE-145^shCtrl^, HFE-145^shNKX6.3#1^, HFE-145^shNKX6.3#2^, PSEC^shCtrl^, and PSEC^shNKX6.3^ cells. All data are presented as mean ± SD of three independent experiments. Asterisks indicates ** *p* < 0.01; *** *p* < 0.001; **** *p* < 0.0001

We investigated the increase in cells exhibiting DNA content that exceeds the 4 N threshold, which indicates disrupted cell cycle control. Depletion of NKX6.3 resulted in significant genomic instability and abnormal cell division, as evidenced by elevated levels of aneuploidy. Microscopic examinations revealed an increase in abnormal mitotic figures in NKX6.3-depleted HFE-145 and PSEC cells compared to control cells, with irregular α-tubulin/DAPI staining indicating compromised mitotic fidelity (Fig. 3a). Additionally, chromosome bridges were significantly more frequent in NKX6.3-depleted cells (Fig. 3b), underscoring the role of NKX6.3 in maintaining chromosomal integrity. Quantification of structural deformities and chromatin condensation further confirmed these disturbances, revealing notable differences between NKX6.3-silenced and control cells (Fig. 3c and d). Furthermore, NKX6.3 depletion upregulated the expression of mitotic spindle formation proteins, including PLK1, PLK2, AurkA, TPX2, BubR1, MAD1, and MAD2 (Supplementary Fig. S1). These findings identify NKX6.3 as a crucial regulator of chromosomal stability, which has significant implications for understanding the molecular mechanisms underlying the development of GC.

### Deciphering the molecular drivers of NKX6.3-associated cellular alterations via sequencing analysis

**Fig. 4:**
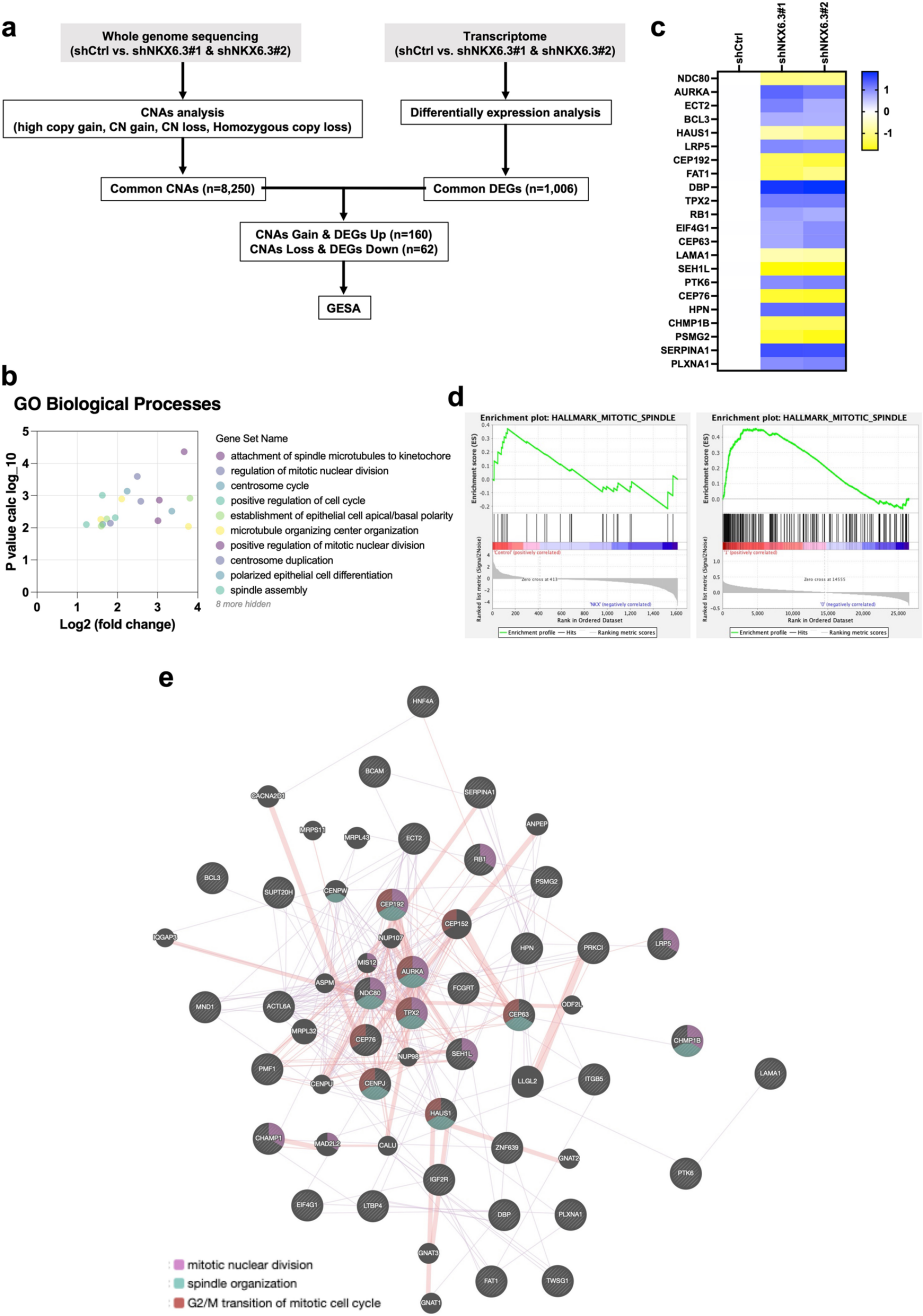
Molecular insights into NKX6.3-associated cellular alterations revealed by sequencing analysis. **a** GSEA illustrating the molecular upheavals underlying cellular irregularities induced by NKX6.3 depletion. **b** GO analysis identifies biological processes significantly altered by NKX6.3 depletion. The table displays selected GO terms with their corresponding log2 fold change and adjusted p-value. **c** Heatmap depicting expression changes of key mitotic spindle-associated genes in HFE-145^shCtrl^, HFE-145^shNKX6.3#1^ and HFE-145^shNKX6.3#2^ cells. Genes like *AurkA* and *TPX2* show significant upregulation in NKX6.3-depleted cells. **d** GSEA enrichment plots for the HALLMARK_MITOTIC_SPINDLE gene set across experimental data and TCGA_STAD dataset. Both datasets show significant positive enrichment, indicating the conserved role of identified genes in mitotic spindle function. **e** Gene network analysis revealing central hub genes, such as *AurkA* and *TPX2*, crucial to mitotic nuclear division, spindle assembly, and the G2/M phase transition

In our study aimed at understanding the molecular causes of abnormal mitotic figures and genomic discrepancies in NKX6.3-deficient HFE-145 cells, we conducted a comprehensive sequencing project comparing HFE-145^shCtrl^ with HFE-145^shNKX6.3#1^ and HFE-145^shNKX6.3#2^ cells. We identified 8,250 CNAs and 1,006 DEGs, revealing complex interactions between genomic and transcriptomic levels. Notably, 160 CNA gains correlated with upregulated DEGs, while 62 CNA losses were associated with downregulated DEGs, which may elucidate the observed phenotypic anomalies. GSEA provided insights into the molecular changes induced by NKX6.3 suppression (Fig. 4a, Supplementary Fig. S2, and Supplementary Table S3). Gene ontology (GO) enrichment analysis revealed key processes such as ‘spindle microtubules to kinetochore’, ‘regulation of mitotic nuclear division’, and ‘centrosome cycle’, aligning with observed atypical mitotic occurrences and chromosomal changes (Fig. 4b and Supplementary Table S4). This underscores the impact of NKX6.3 on cellular homeostasis and genomic stability. Profiling of mitotic spindle-associated genes demonstrated increased expression of AurkA and TPX2 in NKX6.3-depleted cells, indicating a compensatory response (Fig. 4c). GSEA confirmed the significance of these genes in mitotic spindle mechanics, showing strong enrichment in both our dataset and the TCGA_STAD dataset (Fig. 4d and Supplementary Table S5, S6). Network analysis identified AurkA and TPX2 as central hubs in cell cycle regulation, suggesting their potential as therapeutic targets (Fig. 4e and Supplementary Table S7).

### The genetic impact of NKX6.3 depletion

We decreased NKX6.3 expression and analyzed the genetic landscape using whole genome sequencing. A comparison of modified cells to their unaffected counterparts revealed 120 genetic sites with common allelic imbalances (AIs) and loss of heterozygosity (LOH) (Supplementary Fig. S3a and Supplementary Table S8), as well as 1,006 genes exhibiting differential expression. The integration of these datasets identified 40 sites with both genetic imbalance and altered expression, along with 7 sites demonstrating LOH and differential expression (Supplementary Fig. S3b and Supplementary Table S9). GSEA of the REACTOME pathway highlighted significant variations, particularly in ‘Axon guidance’ and ‘Semaphorin interaction’ (Supplementary Fig. S3c). Overlapping gene sets suggested meaningful changes in expression. NKX6.3-depleted cells exhibited increased gene expression in AIs but decreased expression in LOH patterns (Supplementary Fig. S3d and Supplementary Table S9), indicating the role of NKX6.3 in maintaining genomic integrity.

### NKX6.3 deficiency drives amplification of AurkA and TPX2 genes in gastric carcinogenesis

**Fig. 5:**
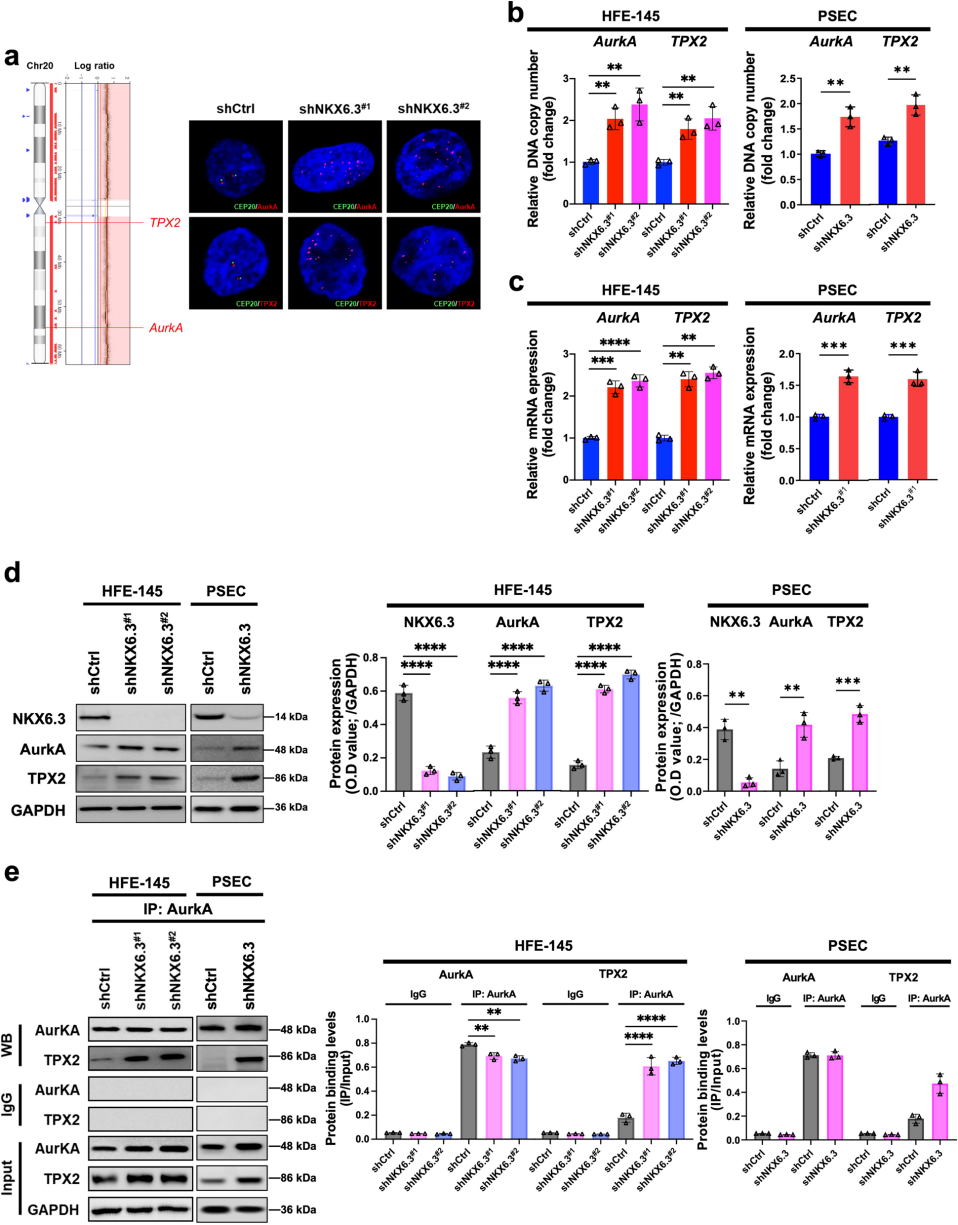
NKX6.3 depletion induces amplification and dysregulation of AurkA and TPX2 genes.** a** Genomic amplification of AurkA and TPX2 genes on chromosome 20 in NKX6.3-depleted HFE-145 cells as evidenced by DNA copy number analysis and FISH. **(b **and** c)** Real-time PCR analysis of AurkA and TPX2 DNA copy number (**b**) and mRNA expression (**c**) in NKX6.3-depleted HFE-145 and PSEC cells. cells compared to control cells. **d** Western blot and ELISA analysis demonstrating elevated protein levels of AurkA and TPX2 in both NKX6.3 depleted cells. **e** Protein-protein interactions between NKX6.3, AurkA, and TPX2 proteins were evaluated by co-IP and ELISA analysis. The depletion of NKX6.3 in both cell types results in an increased binding affinity between AurkA and TPX2. All data are presented as mean ± SD of three independent experiments. Asterisks indicates ** *p* < 0.01; *** *p* < 0.001; **** *p* < 0.0001

In our extensive analysis of hub-gene networks, we found that *AurkA* and *TPX2* are key regulators of mitotic spindle assembly. This discovery led us to further investigate the genomic and proteomic landscapes of these genes after NKX6.3 depletion. Our findings revealed a significant increase in the DNA copy number of these genes on chromosome 20 in the NKX6.3-depleted HFE-145 cells (shNKX6.3^#1^ and shNKX6.3^#2^) (Fig. 5a). This genomic amplification, which is a characteristic of extensive cellular reprogramming, was confirmed through FISH, showing increased numbers of copies (Fig. 5a).

Subsequent real-time PCR and Western blot analyses revealed an increase in DNA copy number, mRNA, and protein expression of these essential genes (Fig. 5b-d) in NKX6.3 depleted HFE-145 and PSEC cells, indicating a series of genetic modifications. Furthermore, the silencing of NKX6.3 in these cells resulted in a noticeable increase in the binding affinity between AurkA and TPX2 proteins, as demonstrated by Western blot analysis (Fig. 5e). These results underscore the essential role of NKX6.3 in regulating key mitotic factors and preventing chromosomal abnormalities.

### Regulatory impact of NKX6.3 on AurkA and TPX2 expression and mitotic integrity in Xenograft mouse models

**Fig. 6:**
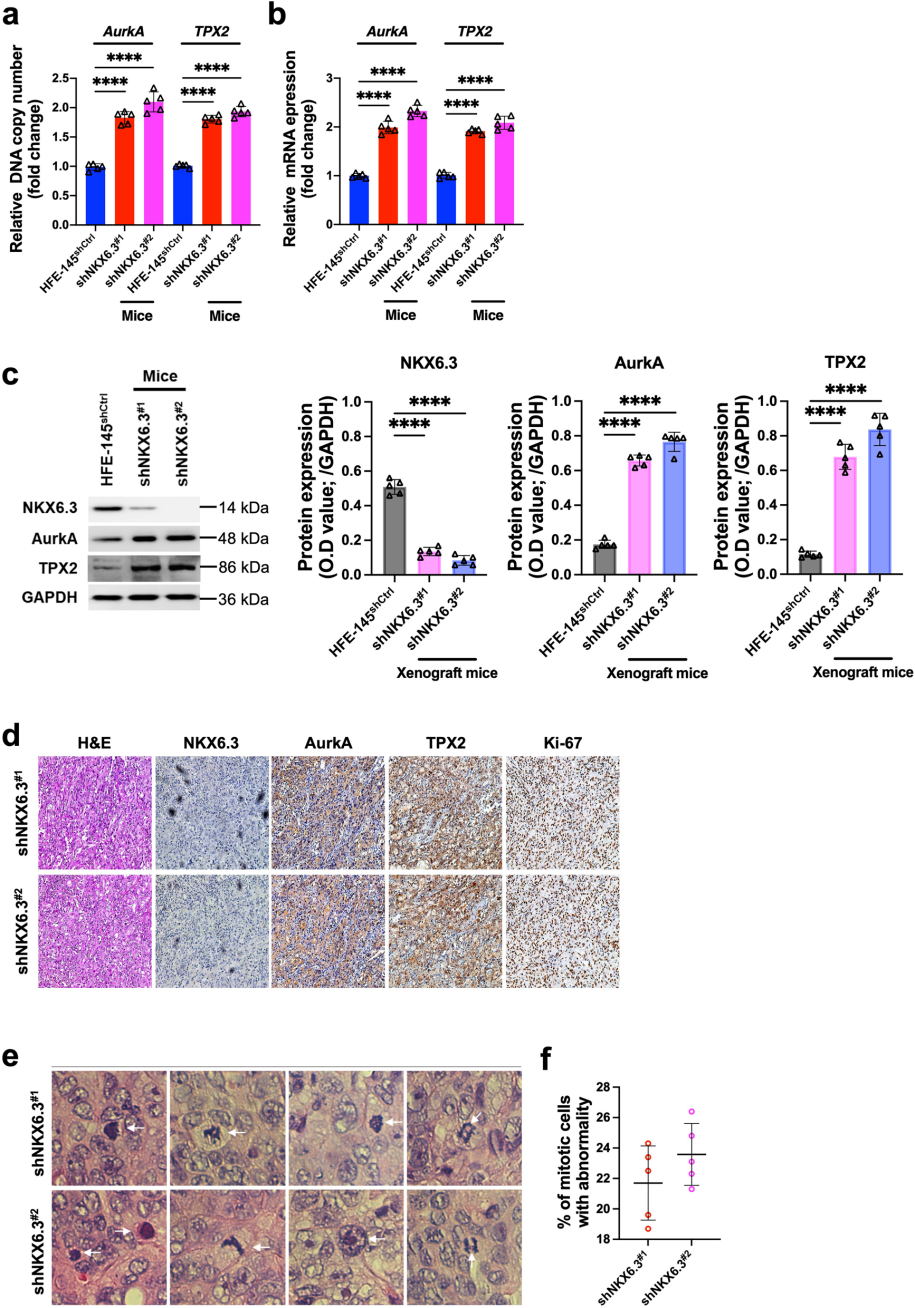
Regulatory Impact of NKX6.3 on AurkA and TPX2 Expression and Mitotic Integrity in Xenograft Mouse Models. **a** and **b** Relative DNA copy number (**a**) and mRNA expression (**b**) levels of AurkA and TPX2 in NKX6.3 depleted cells injected xenograft mouse models compared to HFE-145^shCtrl^ cells. **c** Western blot and ELISA analysis showing protein expression levels of AurkA and TPX2 in HFE-145^shNKX6.3^ xenograft mice and HFE-145^shCtrl^ cells. **d** Histological examination by H&E staining revealing cellular morphology changes and immunohistochemical analysis for NKX6.3, AurkA, TPX2, and Ki-67 markers highlighting regulatory effects. **e** Representative H&E staining of xenograft tumor sections from NKX6.3-depleted cells injected tumor nodule. **f** Quantification of aberrant mitotic figures in NKX6.3-depleted cells injected xenograft tumor sections. All data are presented as mean ± SD of three independent experiments. Asterisk indicates **** *p* < 0.0001

Consistent with our in vitro results, experiments conducted using a xenograft mice model have validated the amplification of AurkA and TPX2 genes. Since tumors did not form in xenograft mice injected with HFE-145^shCtrl^ cells [16], we compared the tumors generated in the NKX6.3 depleted groups to HFE-145^shCtrl^ cells for a comprehensive analysis. The NKX6.3 depleted groups exhibited significantly higher relative DNA copy number and mRNA expression levels of these genes compared to the HFE-145^shCtrl^ cells (Fig. 6a and b). Western blot analysis showed a significant increase in protein expression levels of these genes in HFE-145^shNKX6.3^ xenograft mice, in contrast to their HFE-145 counterparts (Fig. 6c). Histological examination, using H&E staining, revealed significant changes in cellular morphology when NKX6.3 was silenced. Immunohistochemical analysis of NKX6.3, AurkA, TPX2, and Ki-67 markers further demonstrated the regulatory effects of NKX6.3. Different levels of protein expression were observed in shNKX6.3^#1^ and shNKX6.3^#2^ treatments, particularly in AurkA and TPX2, which play a crucial role in mitotic spindle formation, as well as Ki-67, a marker for cellular proliferation (Fig. 6d). The data emphasizes the important role of NKX6.3 in regulating the expression of key proteins that are involved in both cell division and tumor progression.

Histological examination of H&E-stained sections from mice transplanted with NKX6.3-depleted cells revealed a clear anaplastic phenotype. The sections showed structural abnormalities that suggest significant disruptions in cellular differentiation and accurate cell division. Importantly, the quantified data showed a significant increase in abnormal mitotic activity, ranging from 22 to 30%, in the NKX6.3-depleted groups (Fig. 6e and f). This disparity highlights the essential role of NKX6.3 in protecting the integrity of mitotic processes in gastric epithelial cells.

#### Association of NKX6.3 expression with AurkA and TPX2 in human gastric cancers

**Fig. 7:**
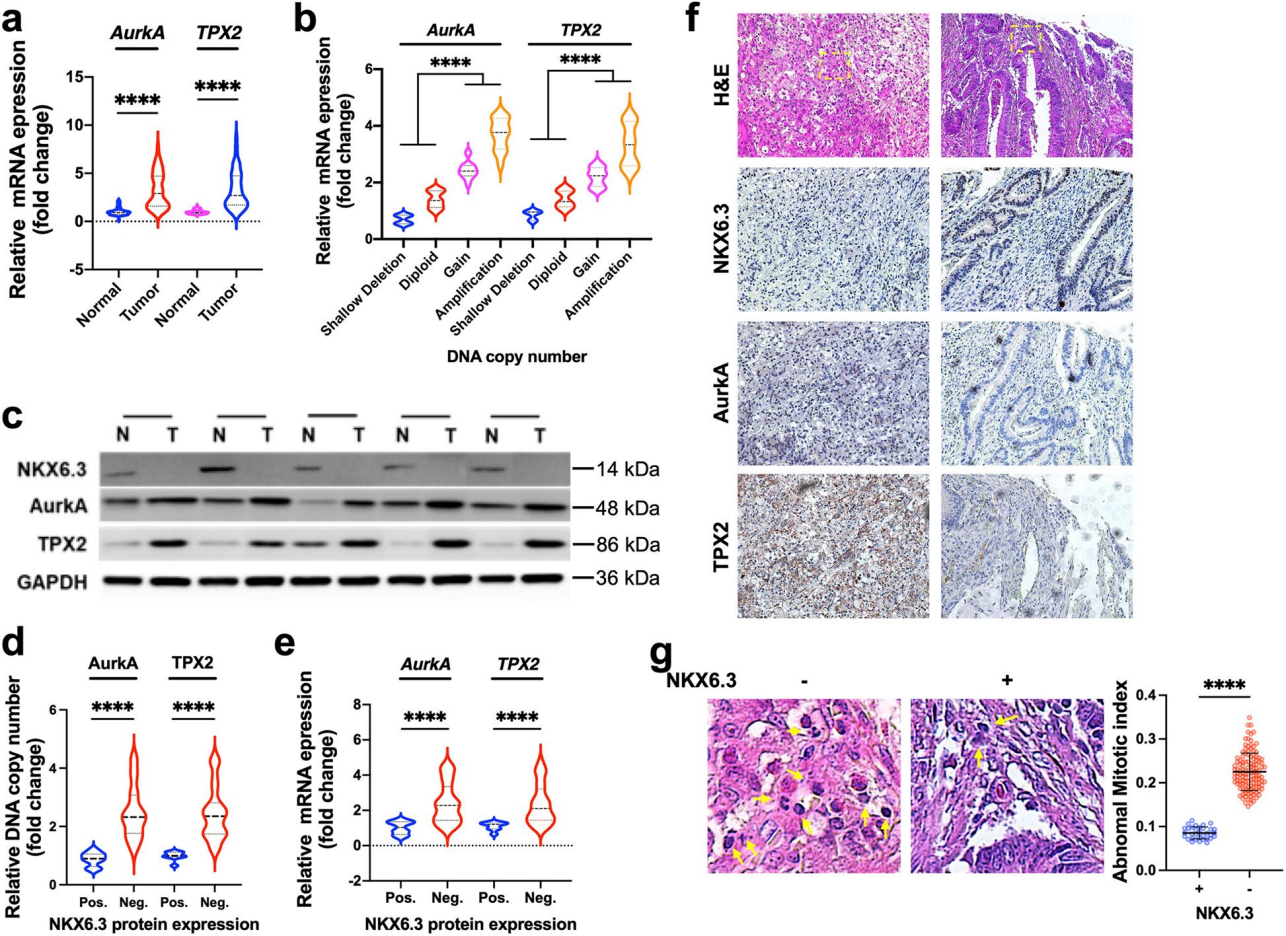
NKX6.3 expression is inversely correlated with AurkA and TPX2 overexpression and mitotic abnormalities in gastric cancer.** a** Upregulation of AurkA and TPX2 mRNA expression in human gastric cancers (*n* = 65). **b** Correlation between DNA copy number variations and mRNA expression of AurkA and TPX2 genes. Y-axis denotes relative mRNA expression (fold change), X-axis categorizes DNA copy number status (Shallow Deletion, Diploid, Gain/Amplification). **c-e** Inverse correlation between NKX6.3 protein expression and AurkA/TPX2 protein levels (**c**), DNA copy number (**d**), and mRNA expression (**e**) in gastric cancer tissues. **f** Immunohistochemical staining showing enhanced protein levels of AurkA and TPX2 in NKX6.3 negative gastric cancer tissues compared to NKX6.3 positive tissues. **g** Comparative analysis of mitotic integrity via H&E staining revealed a decrease in abnormal mitotic figures and the abnormal mitotic index in NKX6.3-positive gastric cancer tissues. All data are presented as mean ± SD of three independent experiments. Asterisk indicates **** *p* < 0.0001

Our investigation focused on the impact of depleting NKX6.3 on the DNA copy number and expression of two important genes, AurkA and TPX2, which are crucial for regulating mitosis and involved in centrosome maturation, segregation, mitotic entry, and spindle assembly. In a group of 65 human GCs, we found a significant increase in the expression of AurkA and TPX2, emphasizing their significance in a tumorous context (Fig. 7a). Furthermore, we analyzed the relationship between CNAs and mRNA expression of AurkA and TPX2, which are integral to regulating mitosis. The scatter plot visually represents this relationship, with the Y-axis indicating the relative mRNA expression as a fold change and the X-axis categorizing the DNA copy number status as Shallow Deletion, Diploid, and Gain/Amplification. Importantly, both the AurkA and TPX2 genes showed a substantial increase in expression in the Gain/Amplification category (Fig. 7b). This positive correlation suggests that gene amplification contributes to the overexpression of genes in the pathogenesis of GC. Additionally, the expression of NKX6.3 protein was found to be inversely correlated with the levels of AurkA and TPX2 protein, DNA copy number, and mRNA levels. This demonstrates the complex regulatory role of NKX6.3 in GC (Fig. 7c-e). In our examination of GC specimens, we used immunohistochemical staining to analyze protein expression profiles. The analysis revealed a clear difference between tissues that were negative for NKX6.3 and those that were positive, which was further supported by staining for AurkA and TPX2. The staining showed higher levels of these proteins in NKX6.3 negative samples (Fig. 7f). In our investigation of the regulatory effects of NKX6.3 on mitotic integrity in GC, we conducted a comparative analysis utilizing H&E staining techniques. Our results showed a significant decrease in abnormal mitotic figures in GC tissues that expressed NKX6.3 compared to those that did not. This was visually and quantitatively confirmed by the reduced abnormal mitotic index in NKX6.3 positive samples (Fig. 7g). These findings underscore the critical role of NKX6.3 in maintaining mitotic integrity and preventing the progression of genomic instability in GC.

## Discussion

In this study, we investigated the impact of NKX6.3 depletion on cellular dynamics, mitotic integrity, and genomic stability in gastric epithelial cells. Our findings demonstrate that NKX6.3 plays a crucial role in regulating cell cycle progression, ensuring mitotic fidelity, and maintaining genomic stability. Notably, the loss of NKX6.3 resulted in accelerated cell cycle progression, an increase in abnormal mitotic figures, and CIN, as evidenced by elevated DNA content and structural aberrations. Additionally, we observed increased expression of key mitotic regulators, AurkA and TPX2, in NKX6.3-deficient cells, both in vitro and in xenograft mouse models. These results position NKX6.3 as a vital regulator of genomic integrity and suggest its potential as a biomarker for assessing mitotic dysfunction in gastric carcinogenesis.

Accelerated cell cycle progression, as observed in NKX6.3-depleted cells, aligns with established models of cancer biology, where dysregulated cell cycle dynamics are frequently associated with tumorigenesis [17–19]. Previous studies have demonstrated that aberrant expression of cyclins and cyclin-dependent kinases (CDKs) can lead to unchecked cell cycle progression, compromising the coordination of mitotic events [3, 19–22]. This misregulation is a well-recognized driver of CIN, an important characteristic of many cancers, including GC, which manifests through chromosomal missegregation and a defective DNA damage response during mitosis [18, 23–26]. Our data indicate that the loss of NKX6.3 results in the upregulation of key cell cycle proteins, such as cyclins and CDKs, accelerating the G1/S transition and disrupting mitotic timing. This acceleration predisposes cells to mitotic errors, abnormal mitotic figures, and further genomic instability, which are hallmarks of malignant transformation. Thus, NKX6.3 appears to function as a critical checkpoint regulator that ensures proper cell cycle progression and mitotic fidelity. CIN, a characteristic feature of cancer cells with accelerated cell cycle progression, arises from defects in mitotic checkpoints and the machinery responsible for chromosome segregation [27–30]. The compromised function of mitotic checkpoints and DNA damage response pathways further amplifies the accumulation of errors during mitosis, perpetuating the cycle of CIN and abnormal mitotic figures [28, 29, 31–33].

One of the significant findings of our study is the association between NKX6.3 depletion and the overexpression of AurkA and TPX2. These mitotic regulators are crucial for proper spindle formation and chromosome segregation. AurkA and TPX2 have been extensively studied for their roles in mitosis and genomic stability. AurkA facilitates centrosome maturation and spindle assembly, while TPX2 regulates spindle dynamics and microtubule nucleation [34–47]. Their overexpression in various cancers, including GC, has been correlated with increased CIN and poor prognosis [42–49]. Our results confirm that NKX6.3 modulates the expression of these mitotic regulators. The dysregulation of AurkA and TPX2 following NKX6.3 depletion leads to persistent mitotic errors, such as lagging chromosomes and chromosomal bridges. These mitotic aberrations contribute to the increased genomic instability observed in NKX6.3-deficient cells, suggesting that NKX6.3 may serve as a potential diagnostic marker for assessing mitotic integrity in GC. Despite these intriguing findings, the significant limitation of our study is the uncertainty regarding the mechanism by which NKX6.3 regulates the expression of AurkA and TPX2.​ Further studies are strongly necessary to clarify the molecular dynamics and to identify potential target genes that NKX6.3 may regulate, thereby elucidating its precise role in modulating AurkA and TPX2 expression.

From a diagnostic pathology perspective, our findings underscore the potential role of NKX6.3 as a biomarker for genomic stability and mitotic regulation in GC. The altered expression of NKX6.3 may serve as a diagnostic tool for identifying high-risk patients exhibiting early mitotic dysregulation and genomic instability, thereby facilitating early detection and risk stratification. Furthermore, the dysregulation of AurkA and TPX2, adds further diagnostic value, as these proteins are frequently overexpressed in various cancers and are linked to tumor progression and therapeutic resistance. Monitoring the expression of these mitotic regulators alongside NKX6.3 status may provide a novel approach for the diagnosis and prognosis of GC.

Furthermore, our study suggests that targeting NKX6.3 or its downstream pathways could provide new therapeutic opportunities. Restoring NKX6.3 function or modulating the expression of AurkA and TPX2 may help prevent the aberrant mitotic progression and CIN that drive GC development. Further research into the NKX6.3 regulatory network could reveal additional molecular targets for therapeutic intervention, thereby advancing precision medicine approaches in the treatment of GC.

## Conclusions

Our study provides new insights into the critical role of NKX6.3 in regulating mitotic integrity, genomic stability, and cellular proliferation in gastric epithelial cells. When this regulation is disrupted, it contributes to the acceleration of cell cycle progression and the formation of abnormal mitotic figures. The causal relationship between NKX6.3 depletion, cell cycle dysregulation, and the subsequent amplification of AurkA and TPX2 underscores the complex molecular pathways involved in CIN and tumor progression. By elucidating these mechanisms, our findings establish a foundation for future research focused on developing targeted therapies that restore genomic stability and inhibit the progression of GC.

In summary, our findings deepen our understanding of GC biology and suggest potential strategies for developing innovative therapeutic interventions. Further investigation into NKX6.3 and its regulatory network holds promise for enhancing patient care and outcomes in the management of GC.

## Supplementary Information

Supplementary Material 1.

Supplementary Material 2.

Supplementary Material 3.

Supplementary Material 4.

Supplementary Material 5.

Supplementary Material 6.

Supplementary Material 7.

Supplementary Material 8.

Supplementary Material 9.

Supplementary Material 10.

## Data Availability

No datasets were generated or analysed during the current study.

## Abbreviations

GC: gastric cancer
CIN: chromosomal instability
NKX6.3: NK6 homeobox 3
WGS: whole-genome sequencing
ELISA: enzyme-linked immunosorbent assay
DEGs: Differentially expressed genes
GSEA: gene set enrichment analysis
FISH: fluorescence in situ hybridization
CNAs: copy number alterations
IHC: immunohistochemistry
AIs: allelic imbalances
LOH: loss of heterozygosity
AurkA: Aurora Kinase A
TPX2 TPX2: Microtubule Nucleation Factor
CDKs: Cyclin-dependent kinases
PSEC: Human primary stomach epithelial cells
