# Supplementary material for: NKX6.3 modulation of mitotic dynamics and genomic stability in gastric carcinogenesis
Source: Cell Commun Signal. 2025 Jan 20;23:35. doi: 10.1186/s12964-025-02030-4 (PMC11748348; doi:10.1186/s12964-025-02030-4)
Supplement: Supplementary file 1 — Supplementary Material 1. [file 12964_2025_2030_MOESM1_ESM.docx]

**Table S1. List of primer sequences used for real-time qPCR**

| Experiments | Gene | Sequence | |
| --- | --- | --- | --- |
| real-time qPCR for CNAs | AurkA | Forward | GGACGGTTCTTCTGGAGCTT |
|  |  | Reverse | GCGAGACTTCGTCTCAAAACA |
|  | TPX2 | Forward | AGGGGCCCTTTGAACTCTTA |
|  |  | Reverse | TGCTCTAAACAAGCCCCATT |
|  | γ-Actin | Forward | AGTCGAAGCGTGGTATCCT |
|  |  | Reverse | ACTTGGGGTTGATGGGAG |
| real-time qPCR for mRNA expression | AurkA | Forward | TTTTGTAGGTCTCTTGGTATGTG |
|  |  | Reverse | GCTGGAGAGCTTAAAATTGCAG |
|  | TPX2 | Forward | CGAAAGCATCCTTCATCTCC |
|  |  | Reverse | TCCTTGGGACAGGTTGAAAG |
|  | GAPDH | Forward | GCGACACCCACTCCTCCACC |
|  |  | Reverse | GAGGTCCACCACCCTGTTGC |
