# Supplementary material for: NKX6.3 modulation of mitotic dynamics and genomic stability in gastric carcinogenesis
Source: Cell Commun Signal. 2025 Jan 20;23:35. doi: 10.1186/s12964-025-02030-4 (PMC11748348; doi:10.1186/s12964-025-02030-4)
Supplement: Supplementary file 2 — Supplementary Material 2. [file 12964_2025_2030_MOESM2_ESM.docx]

**Table S2. List of antibodies used in this study.**

| **Protein** | **Company** | **Cat No.** |
| --- | --- | --- |
| NKX6.3 | Atlas Antibodies | HPA042790 |
| CDK4 | Abcam | ab137675 |
| CDK6 | Abcam | ab151247 |
| Cyclin D | Cell signaling | 2922 |
| p-p53 (Ser 15) | Cell signaling | 9286 |
| p53 | Cell signaling | 2524 |
| p21 | Cell signaling | 2947 |
| p16 | Cell signaling | 80772 |
| CDK2 | Santa Cruz | sc-6248 |
| Cyclin E | Santa Cruz | sc-247 |
| Cdc25c | Abcam | ab32444 |
| Cdc25A | Santa Cruz | sc-7389 |
| p-Cdc2 (Tyr 15) | Santa Cruz | sc-7989 |
| CDK1 | Abcam | ab18 |
| Wee1 | Cell signaling | 4936 |
| CDT1 | Cell signaling | 3386 |
| Cyclin B | Abcam | ab2949 |
| GAPDH | Santa Cruz | sc-32233 |
| PLK1 | Abcam | ab17056 |
| PLK2 | Cell signaling | 14812 |
| AurKA | Abcam | ab13824 |
| TPX2 | Abcam | ab32795 |
| BubR1 | Abcam | ab28193 |
| MAD1 | Santa Cruz | sc-47746 |
| MAD2 | Santa Cruz | sc-47747 |
| a-Tubulin | Cell signaling | 2144 |
| Ki-67 | Cell signaling | 12202 |
