# Supplementary material for: NKX6.3 modulation of mitotic dynamics and genomic stability in gastric carcinogenesis
Source: Cell Commun Signal. 2025 Jan 20;23:35. doi: 10.1186/s12964-025-02030-4 (PMC11748348; doi:10.1186/s12964-025-02030-4)
Supplement: Supplementary file 10 — Supplementary Material 10. [file 12964_2025_2030_MOESM10_ESM.docx]

**Supplementary Figure S1**

**Expression of mitotic spindle formation related proteins by NKX6.3.** Depletion of NKX6.3 increased PLK1, PLK2, AurkA, TPX2, BubR1, MAD1, and MAD2 expression

**Supplementary Figure S2**

**Transcriptome analysis of HFE-145 cells after NKX6.3 depletion.** (**a)** Heatmap showing differential gene expression in HFE-145 cells post NKX6.3 depletion, with color intensity representing expression levels. (**b)** Volcano plots indicating significant gene expression changes between control and NKX6.3-depleted cells, with upregulated genes in orange and downregulated genes in blue. (**c)** Venn diagrams depicting the overlap of upregulated and downregulated genes between HFE-145^shNKX6.3#1^ and HFE-145^shNKX6.3#2^ cells. (**d)** Scatter plot for REACTOME pathway enrichment analysis, with dot size denoting gene count and color intensity showing p-value significance. (**e)** Heatmap of specific genes involved in mitotic spindle, comparing expression levels across HFE-145^shCtrl^, HFE-145^shNKX6.3#1^ and HFE-145^shNKX6.3#2^ cells

**Supplementary Figure S3**

**Genetic consequences of NKX6.3 depletion revealed by whole genome sequencing in HFE-145 gastric epithelial cells.** (**a)** Comparison of genetic landscape alterations, such as Allelic imbalance and LOH, between NKX6.3-depleted cells and HFE-145^shCtrl^ using whole genome sequencing. (**b)** Summary of common allelic imbalances, loss of heterozygosity (LOH), and differential gene expression identified post-NKX6.3 depletion. (**c)** Gene Set Enrichment Analysis (GSEA) highlighting affected biological pathways, with emphasis on key gene sets such as 'Axon guidance' and 'Semaphorin interaction'. (**d)** A scatter plot illustrates gene expression levels in allelic imbalance and LOH patterns, showcasing contrasting expression profiles between HFE-145^shCtrl^ and NKX6.3-depleted cells. Asterisks indicates ** p < 0.01; *** p < 0.001; **** p < 0.0001
